# Residual pattern of primary tumor and lymph node in ESCC treated with nCRT with or without pembrolizumab: an analysis from a prospective cohort

**DOI:** 10.3389/fimmu.2025.1700400

**Published:** 2025-10-22

**Authors:** Xuan Han, Wei-Xiang Qi, Shu-Yan Li, Huan Li, Jia-Yi Chen, Sheng-Guang Zhao

**Affiliations:** ^1^ Department of Radiation Oncology, Ruijin Hospital, Shanghai Jiaotong University School of Medicine, Shanghai, China; ^2^ Shanghai Key Laboratory of Proton-therapy, Shanghai, China

**Keywords:** esophageal squamous cell carcinoma, neoadjuvant immunochemoradiotherapy, neoadjuvant chemoradiotherapy, primary tumor residual, lymph node spread

## Abstract

**Background:**

Neoadjuvant chemoradiotherapy (nCRT) is recommended as the standard of care for locally advanced esophageal squamous cell carcinoma (ESCC). Adding immunotherapy to nCRT (nICRT) has gained attention in clinical practice. We evaluated the differences in clinicopathologic outcomes and the patterns of lymph node metastasis in patients receiving nCRT and nICRT for locally advanced ESCC.

**Methods:**

A total of 208 ESCC patients who completed transthoracic esophagectomy after neoadjuvant treatment were enrolled. Clinicopathologic parameters and the rates of lymph node metastasis in each station classified using both the eighth edition of the American Joint Committee on Cancer (AJCC) esophageal cancer staging system and the 11th edition of the Japanese Classification of Esophageal Cancer (JCEC) standard were recorded and evaluated.

**Results:**

The rates of pathological complete response (pCR) and major pathological response (MPR) were 44.9% in nICRT vs. 37.0% in nCRT (*p* = 0.263) and 79.5% in nICRT vs. 65.4% in nCRT (*p* = 0.024), respectively. The common sites of lymph node metastasis after neoadjuvant treatment were station 112pulL (8.3%), followed by station 104L (4.9%), station 7 (4.5%), and station 3a (4.3%), according to the 11th JCEC standard. Compared with nCRT, nICRT can significantly reduce the rates of lymph node metastasis in station 2R (0.8% vs. 4.6%, *p* = 0.039) classified using the AJCC system, and those in station 106recR (0.8% vs. 4.6%, *p* = 0.042) and station 20 (0 vs. 12.5%, *p* = 0.030) classified using the JCEC standard.

**Conclusion:**

nICRT followed by surgery may lead to a promising pathological response. For patients with lymph node metastasis in certain regions, nICRT should be considered as a better preoperative treatment option.

## Introduction

Esophageal cancer is the sixth most common cause of cancer-related mortality and ranks seventh in incidence among all malignancies worldwide, with the highest incidence rates in eastern Asia (1). Esophageal squamous cell carcinoma (ESCC) is the predominant histological subtype of esophageal cancer globally, accounting for approximately 90% (2). Curative resection is still a major component of current treatment for ESCC. Based on the results of the ChemorRdiotherapy for Oesophageal cancer followed by Surgery Study (CROSS) (3–5) and neoadjuvant chemoradiotheraoy for esophageal cancer 5010 (NEOCRTEC5010) (6) trials, neoadjuvant chemoradiotherapy (nCRT) followed by esophagectomy is applied as the standard of care for locally advanced ESCC (7). However, there are still 49% patients developing either locoregional or distant recurrence after this regimen, and the 5-year overall survival rate is approximately 47% (4). Novel and effective treatment strategies for locally advanced ESCC to further improve prognosis are desperately needed.

The application of immunotherapy in the neoadjuvant setting of locally advanced ESCC has gained attention in clinical practice (8). The addition of immune checkpoint inhibitors (ICIs) to neoadjuvant chemotherapy, namely, neoadjuvant immunochemotherapy (nICT), has achieved pathological complete response (pCR) rates between 16.7% and 35.3% for locally advanced ESCC patients in ESONICT-1 (9) and ESONICT-2 (10) trials. Other trials have also shown that this combination may have synergistic and greater effects compared with chemotherapy alone (11–14). Given that nCRT is recommended as the first-line strategy for locally advanced ESCC and the radiation-induced enhancement of antitumor efficacy of immunotherapy (15–17), researchers are moving forward with their work to combine ICIs with nCRT, namely, neoadjuvant immunochemoradiotherapy (nICRT), to attempt to further improve the efficacy of the existing neoadjuvant treatment and expand clinicians’ options. Zhu et al. found that the combination of pembrolizumab and nCRT as treatment for gastroesophageal junction adenocarcinoma achieved an improved pCR rate in patients with Programmed Cell Death Ligand 1 (PD-L1) Combined Positive Score (CPS) ≥ 10 compared with those with PD-L1 CPS < 10 (50% vs. 13.6%, *p* = 0.046) (18). The PERFECT trial conducted by van den Ende et al. showed that the addition of atezolizumab to nCRT revealed a pCR rate of 25% but without a survival benefit in patients with resectable esophageal adenocarcinoma (19). As for nICRT in locally advanced ESCC, the single-armed PALACE-1 trial conducted by our institute confirmed the safety and activity of preoperative pembrolizumab combined with nCRT with a preliminary 55.6% (10/18) pCR rate (20), and a subsequent multicenter single-arm PALACE-2 trial investigating the efficacy is ongoing to further confirm the safety of nICRT (21).

Postoperative pathological response and staging (ypTNM) are closely correlated with patients’ risk of recurrence. Previous studies have demonstrated that the depth of primary tumor residual (ypT) and the number of metastatic lymph nodes (ypN) were critical predictors for survival in patients with esophageal cancer receiving neoadjuvant treatment (22). Patients achieving pCR were more likely to have a better prognosis, while the 5-year survival rates of patients with ypT3+ or ypN+ were only 30% and 25.6%, respectively (23). Furthermore, ESCC has an earlier propensity for lymphatic spread since the esophageal wall has a rich lymphatic drainage network, and the lymphatic vessels of the thoracic esophagus can drain to the cervical, thoracic, and abdominal lymph node stations. This characteristic brings about difficulty in defining the proper sentinel lymph nodes (24). The nICRT strategy was given high expectations in disease control, but it has not been determined yet whether the pattern of primary tumor residual and lymph node spread in locally advanced ESCC patients treated with the nICRT regimen and whether there are differences from the traditional nCRT treatment. Moreover, there are currently two mainstream standards classifying lymph node stations in esophageal cancer applied by clinicians worldwide, namely, the eighth edition of the American Joint Committee on Cancer (AJCC) esophageal cancer staging system (25) and the 11th edition of Japanese Classification of Esophageal Cancer (JCEC) standards (26, 27). Considering that precise lymph node dissection is crucial to improve prognosis, it is necessary to evaluate the pattern of lymph node spread based on both these standards to cater to clinicians with different preferences.

In this study, we analyzed the differences in postoperative pathological characteristics and the pattern of lymph node spread in patients with thoracic ESCC receiving different neoadjuvant treatment strategies, including nCRT and nICRT, from a prospective database to provide a reference for determining the optimal lymph node removal scope and for the selection of treatment regimens.

## Materials and methods

### Patient enrollment

The study was designed retrospectively based on a prospective database (20, 28–31) and was approved by the Ethics Committee of Ruijin Hospital, Shanghai Jiao Tong University School of Medicine, while patients’ informed consent was exempted due to the retrospective nature of the study. Data were collected from patients with previously untreated, locally advanced, and surgically resectable ESCC at our center from April 2019 to December 2023. Pretreatment staging was undertaken for every patient. The key inclusion criteria were 1) pathologically confirmed locally advanced ESCC, 2) ages between 18 and 75 years, 3) Eastern Cooperative Oncology Group performance status score of 0–1, and 4) treated with standardized nCRT with or without pembrolizumab followed by curative surgery. Patients with a history of other malignancies, antitumor treatment, or salvage esophagectomy after definitive chemoradiotherapy were excluded.

### Neoadjuvant strategy

Preoperative treatment was composed of chemotherapy, radiotherapy, and immunotherapy. nCRT was given according to the CROSS regimen (5). The concurrent chemotherapy regimen included carboplatin [area under the curve (AUC) of 2 mg·mL^−1^·min^−1^] and paclitaxel/nab-paclitaxel (50 mg/m^2^), which was administered intravenously once a week for 5 weeks (on days 1, 8, 15, 22, and 29 of the neoadjuvant treatment period). A total radiation dose of 41.4 Gy was given in 23 fractions, with five fractions each week and 1.8 Gy per fraction. Patients in the nICRT group received pembrolizumab concurrently on days 1 and 22 of the neoadjuvant treatment period at a dose of 200 mg in addition to the nCRT regimen. Involved-field irradiation (IFI) was used in the present study, and the detailed methods for countering gross tumor volume (GTV), clinical target volume (CTV), and planning treatment volume (PTV) were described in our previous study (30). The positive metastatic lymph nodes (GTV-nd) were determined according to one of the following criteria: 1) paraoesophageal, tracheoesophageal groove, at the angle of the diaphragm or abdominal lymph nodes with a short diameter ≥0.5 cm; or lymph nodes in other locations with a short diameter ≥1 cm; 2) multiple (≥5) small lymph nodes clustered together; and 3) PET–CT showed lymph nodes with high metabolism and standardized uptake value (SUV) ≥2.5.

### Surgical treatment

After the completion of neoadjuvant treatment, radiology examination (such as contrast-enhanced CT scan and PET–CT), routine laboratory tests, pulmonary function, and electrocardiogram were conducted to reassess the disease and exclude cases with any surgical contraindications. Patients suitable for radical esophagectomy were evaluated by the multidisciplinary team, and the surgical approach was determined by thoracic surgeons. Surgery was arranged 4–6 weeks after the completion of neoadjuvant treatment. For patients with tumors located in the upper third of the esophagus or with cervical lymph node involvement, the McKeown esophagectomy with three-field lymphadenectomy was performed. For patients with tumors located in the middle and lower third of the esophagus, the Ivor Lewis esophagectomy with two-field lymphadenectomy was performed.

### Pathological assessment

Each resected tumor specimen was independently reviewed by two experienced upper gastrointestinal pathologists. Pathological examination included pathological type, tumor length, depth and extent of tumor invasion, resection margin, grade of differentiation, grade of tumor regression, overall and positive lymph nodes dissected, and tumor stage determined by the eighth edition TNM classification of the AJCC for esophageal cancer. R0 resection was defined as a tumor-free resection margin, while R1 and R2 levels were referred to as a resection margin with microscopic and macroscopic residual tumors, respectively. The Chirieac modified tumor regression grade (TRG) system was applied to classify the pathological response of the primary lesion. The extent of residual tumor was divided into four categories: TRG1, no residual carcinoma; TRG2, 1%–10% vital residual carcinoma; TRG3, 11%–50% residual carcinoma; and TRG4, greater than 50% residual carcinoma (32). All dissected lymph nodes were reviewed for their status of metastases, their precise locations, or called stations and were recorded according to two currently mainstream standards, namely, the eighth edition of the AJCC esophageal cancer staging system (25) and the 11th edition of the JCEC standards (26, 27). The complete regression of primary lesion and lymph node metastases was considered pCR, while major pathological response (MPR) was defined as <10% residual viable tumor cells in the primary tumor.

### Statistical analysis

The primary endpoint of our study was pCR, while the secondary endpoints were TRG, MPR, postoperative stage, and specific nodal response. For descriptive statistics, the continuous variable was expressed as a median with its range, while the categorical variable was expressed as frequency with percentage. To compare the differences in baseline characteristics, pathological parameters, and rates of lymph node metastasis in all stations between the nCRT and nICRT groups, a Wilcoxon rank-sum test for continuous variables and Pearson’s chi-squared or Fisher’s exact test for categorical variables were conducted. *p*-Values were two-sided, with a significance level of <0.05 for all analyses. All statistical analyses were performed using the SPSS software for Windows, version 26.0 (IBM Corp., Armonk, NY, USA).

## Results

### Patient characteristics

In our study, a total of 208 patients with locally advanced ESCC who completed transthoracic esophagectomy after neoadjuvant treatment were enrolled. Among them, 81 patients received conventional nCRT, whereas 127 patients received nICRT. The baseline characteristics were comparable between the two groups, including age, gender, primary tumor location, primary tumor length, and clinical staging, which are summarized in **Table 1**.

**Table 1:** Baseline characteristics of two groups of patients.

Characteristics	Level	nCRT (%)	nICRT (%)	*p*-value
Age	<65	31 (38.3)	55 (43.3)	0.472
	≥65	50 (61.7)	72 (56.7)	
Gender	Male	69 (85.2)	106 (83.5)	0.740
	Female	12 (14.8)	21 (16.5)	
Location	Upper	19 (23.5)	23 (18.1)	0.434
	Middle	43 (53.1)	65 (51.2)	
	Lower	19 (23.5)	39 (30.7)	
Tumor length (cm)	<5	28 (34.6)	52 (40.9)	0.357
	≥5	53 (65.4)	75 (59.1)	
cT	2	23 (28.4)	40 (31.5)	0.873
	3	52 (64.2)	77 (60.6)	
	4a	6 (7.4)	10 (7.9)	
cN	0	3 (3.7)	8 (6.3)	0.866
	1	26 (32.1)	41 (32.3)	
	2	39 (48.1)	58 (45.7)	
	3	13 (16.0)	20 (15.7)	
cTNM	II	11 (13.6)	27 (21.3)	0.347
	III	53 (65.4)	73 (57.5)	
	IVA	17 (21.0)	27 (21.3)	

Pearson’s chi-squared or Fisher’s exact test was used. *p*-Values were two-sided.

nICRT, neoadjuvant immunochemoradiotherapy; nCRT, neoadjuvant chemoradiotherapy.

### Pathological response to neoadjuvant treatment

**Figure 1:**
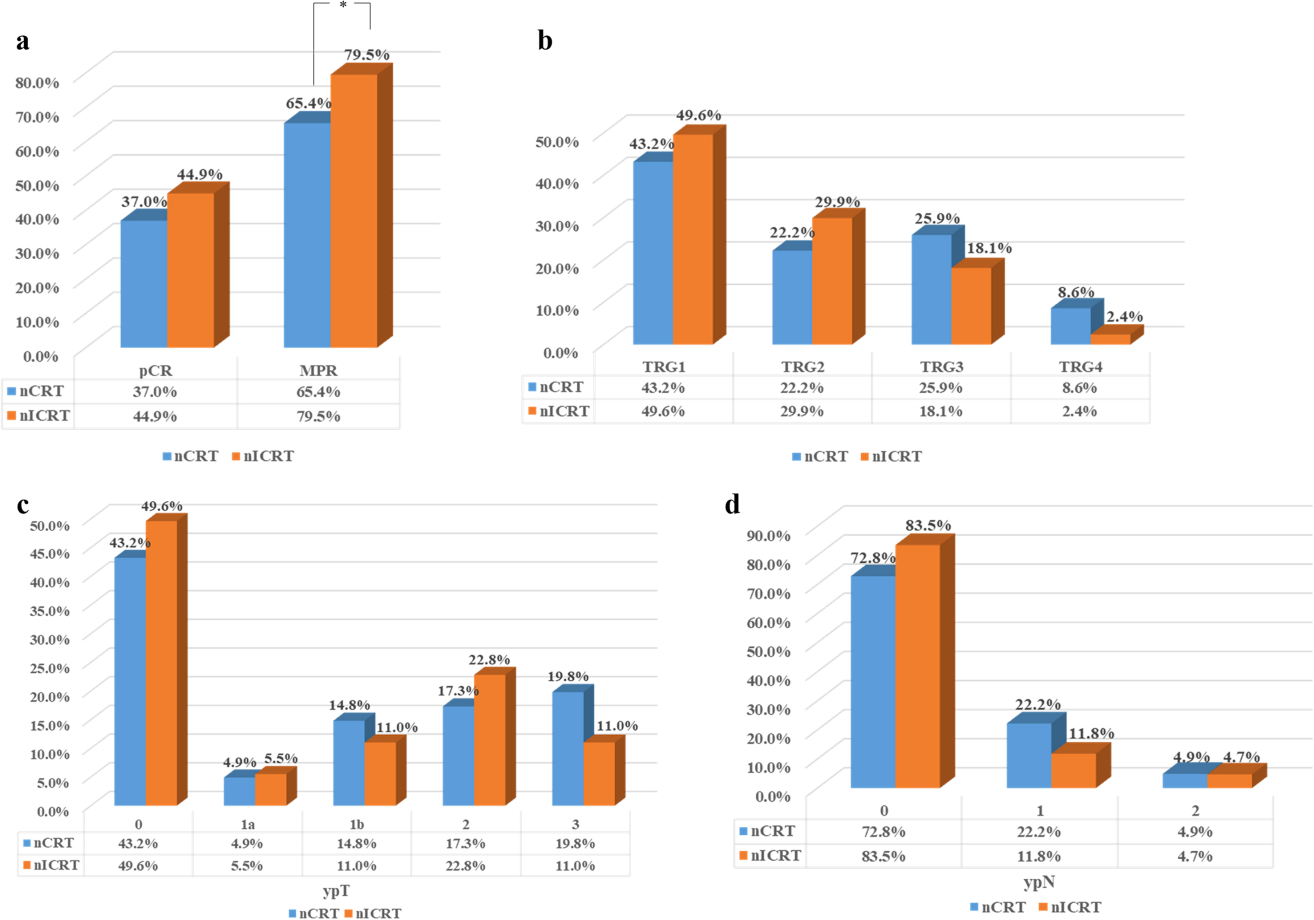
Residual tumor characteristics of all patients in both groups after neoadjuvant therapy. **(a)** Comparisons of the pathological complete response (pCR) and major pathological response (MPR) rates between two groups. **(b)** Tumor regression grade (TRG) score in two groups. Pathological response of primary tumor toward neoadjuvant therapy was evaluated using the Chirieac modified TRG system. **(c)** Comparison of the depth of tumor invasion (ypT) between two groups. **(d)** Comparison of the number of lymph node metastases (ypN) between two groups. *p < 0.05. Pearson’s chi-squared or Fisher’s exact test was used. *p*-Values were two-sided.

We estimated the pathological response to neoadjuvant treatment in the 208 ESCC patients who underwent surgical resection. The results are shown in **Table 2** and **Figure 1**. Patients receiving nICRT showed comparable rates of R0 resection and vascular infiltration to patients receiving conventional nCRT (R0 resection rate: 92.9% vs. 96.3%, *p* = 0.474; vascular infiltration rate: 7.9% vs. 14.8%, *p* = 0.112). However, the rate of nerve invasion in patients receiving nICRT was significantly lower than that of patients receiving nCRT (3.9% vs. 11.1%, *p* = 0.044). Patients in the nICRT group exhibited numerically lower TRG score (*p* = 0.075; **Figure 1b**) and higher pCR rate (44.9% vs. 37.0%, *p* = 0.263; **Figure 1a**), but significantly higher MPR rate (79.5% vs. 65.4%, *p* = 0.024; **Figure 1a**), compared to those in the nCRT group. Compared with nCRT, the nICRT regimen had an advantage in downgrading postoperative TNM stage despite a lack of statistical difference (**Figures 1c, d**).

**Table 2:** Pathological outcomes of two cohorts.

Characteristic	Level	nCRT (%)	nICRT (%)	*p*-value
R0 resection	R1	3 (3.7)	9 (7.1)	0.474
	R0	78 (96.3)	118 (92.9)	
Vascular infiltration	Yes	12 (14.8)	10 (7.9)	0.112
	No	69 (85.2)	117 (92.1)	
Nerve invasion	Yes	9 (11.1)	5 (3.9)	0.044
	No	72 (88.9)	122 (96.1)	
TRG score	1	35 (43.2)	63 (49.6)	0.075
	2	18 (22.2)	38 (29.9)	
	3	21 (25.9)	23 (18.1)	
	4	7 (8.6)	3 (2.4)	
MPR	Yes	53 (65.4)	101 (79.5)	0.024
	No	28 (34.6)	26 (20.5)	
pCR	Yes	30 (37.0)	57 (44.9)	0.263
	No	51 (63.0)	70 (55.1)	
ypT	0	35 (43.2)	63 (49.6)	0.365
	1a	4 (4.9)	7 (5.5)	
	1b	12 (14.8)	14 (11.0)	
	2	14 (17.3)	29 (22.8)	
	3	16 (19.8)	14 (11.0)	
ypN	0	59 (72.8)	106 (83.5)	0.136
	1	18 (22.2)	15 (11.8)	
	2	4 (4.9)	6 (4.7)	
ypTNM	I	52 (64.2)	97 (76.4)	0.328
	II	7 (8.6)	9 (7.1)	
	IIIA	12 (14.8)	12 (9.4)	
	IIIB	10 (12.3)	9 (7.1)	

Pearson’s chi-squared or Fisher’s exact test was used. *p*-Values were two-sided.

nCRT, neoadjuvant chemoradiotherapy; nICRT, neoadjuvant immunochemoradiotherapy; TRG, tumor regression grade; MPR, major pathological response; pCR, pathological complete response.

### Patterns of lymphatic spread in overall and subgroup analyses

Among the 208 patients, 43 (20.7%) had pathologically positive lymph nodes postoperatively, with 22 and 21 patients in the nCRT and nICRT groups, respectively (27.2% vs. 16.5%, *p* = 0.19). We then estimated the metastasis rates of lymph nodes in different locations in the 208 ESCC patients who underwent surgical resection after neoadjuvant therapy. We identified a total of 1,176 and 2,230 lymph nodes, with 37 and 39 metastatic lymph nodes in the nCRT and nICRT groups, respectively (3.1% vs. 1.7%, *p* = 0.0069). The common sites of lymph node metastasis after neoadjuvant treatment were station 112pulL (8.3%, 3/36 nodes positive), followed by station 104L (4.9%, 2/41 nodes positive), station 7 (4.5%, 12/266 nodes positive), and station 3a (4.3%, 10/230 nodes positive) according to the 11th JCEC standard. Based on the eighth of the AJCC esophageal cancer staging system, the metastasis rate of lymph nodes in station 2R in patients receiving nICRT was significantly lower than that of patients receiving nCRT (0.8% vs. 4.6%, *p* = 0.039; **Table 3**). Based on the 11th JCEC standard, metastasis rates of lymph nodes in station 106recR (0.8% vs. 4.6%, *p* = 0.042; **Table 3**) and station 20 (0% vs. 12.5%, *p* = 0.030) had a significant difference between nICRT and nCRT. Among other stations, however, there was no statistical difference in lymph node metastasis rates whether patients received nICRT or nCRT.

**Table 3:** Lymph node metastasis rates of different stations classified using AJCC and JCEC standards among two groups of patients.

AJCC Station	Level	nCRT (%)	nICRT (%)	*p*-value	JCEC Station	Level	nCRT (%)	nICRT (%)	*p*-value
1L	Positive	0 (0.0)	2 (7.4)	0.539	104L	Positive	0 (0.0)	2 (7.4)	0.539
	Negative	14 (100.0)	25 (92.6)			Negative	14 (100.0)	25 (92.6)	
1R	Positive	0 (0.0)	0 (0.0)	NA	104R	Positive	0 (0.0)	0 (0.0)	NA
	Negative	4 (100.0)	20 (100.0)			Negative	4 (100.0)	20 (100.0)	
2L	Positive	3 (3.5)	2 (1.3)	0.472	106pre	Positive	0 (0.0)	0 (0.0)	NA
	Negative	82 (96.5)	157 (98.7)			Negative	3 (100.0)	15 (100.0)	
2R	Positive	6 (4.6)	2 (0.8)	**0.039**	106recL	Positive	3 (3.5)	2 (1.3)	0.472
	Negative	125 (95.4)	247 (99.2)			Negative	82 (96.5)	157 (98.7)	
8U	Positive	3 (4.1)	2 (1.9)	0.671	106recR	Positive	6 (4.6)	2 (0.8)	**0.042**
	Negative	70 (95.9)	104 (98.1)			Negative	124 (95.4)	241 (99.2)	
4L	Positive	1 (2.6)	0 (0.0)	0.141	105	Positive	3 (4.2)	2 (1.9)	0.647
	Negative	38 (97.4)	75 (100.0)			Negative	68 (95.8)	104 (98.1)	
4R	Positive	0 (0.0)	0 (0.0)	NA	106tbL	Positive	0 (0.0)	0 (0.0)	NA
	Negative	19 (100.0)	39 (100.0)			Negative	10 (100.0)	19 (100.0)	
7	Positive	1 (0.7)	4 (1.3)	0.903	106tbR	Positive	0 (0.0)	0 (0.0)	NA
	Negative	150 (99.3)	309 (98.7)			Negative	5 (100.0)	4 (100.0)	
8M	Positive	1 (1.5)	2 (1.3)	0.908	107	Positive	1 (0.7)	4 (1.3)	0.903
	Negative	65 (98.5)	150 (98.7)			Negative	150 (99.3)	309 (98.7)	
8Lo	Positive	3 (3.4)	3 (2.1)	0.842	108	Positive	1 (1.7)	2 (1.4)	0.873
	Negative	85 (96.6)	142 (97.9)			Negative	59 (98.3)	144 (98.6)	
9L	Positive	2 (14.3)	1 (4.5)	0.680	109L	Positive	1 (3.4)	0 (0.0)	0.140
	Negative	12 (85.7)	21 (95.5)			Negative	28 (96.6)	56 (100.0)	
9R	Positive	0 (0.0)	0 (0.0)	NA	109R	Positive	0 (0.0)	0 (0.0)	NA
	Negative	5 (100.0)	13 (100.0)			Negative	13 (100.0)	26 (100.0)	
15	Positive	0 (0.0)	0 (0.0)	NA	110	Positive	3 (3.5)	2 (1.4)	0.561
	Negative	8 (100.0)	23 (100.0)			Negative	83 (96.5)	141 (98.6)	
16	Positive	8 (2.9)	6 (1.2)	0.095	112pulL	Positive	2 (14.3)	1 (4.5)	0.680
	Negative	271 (97.1)	491 (98.8)			Negative	12 (85.7)	21 (95.5)	
17	Positive	8 (5.6)	14 (4.0)	0.425	112pulR	Positive	0 (0.0)	0 (0.0)	NA
	Negative	135 (94.4)	339 (96.0)			Negative	5 (100.0)	13 (100.0)	
18	Positive	0 (0.0)	0 (0.0)	NA	112aoA	Positive	0 (0.0)	1 (12.5)	0.444
	Negative	21 (100.0)	79 (100.0)			Negative	10 (100.0)	7 (87.5)	
19	Positive	0 (0.0)	0 (0.0)	NA	111	Positive	0 (0.0)	0 (0.0)	NA
	Negative	10 (100.0)	21 (100.0)			Negative	8 (100.0)	23 (100.0)	
20	Positive	1 (3.8)	1 (2.7)	1.000	20	Positive	3 (12.5)	0 (0.0)	**0.030**
	Negative	25 (96.2)	36 (97.3)			Negative	21 (87.5)	51 (100.0)	
Total	Positive	37 (3.1)	39 (1.7)	**0.0069**	1	Positive	4 (2.8)	5 (2.0)	0.875
	Negative	1,139 (96.9)	2,291 (98.3)			Negative	139 (97.2)	245 (98.0)	
					2	Positive	1 (0.9)	1 (0.5)	0.693
						Negative	111 (99.1)	195 (99.5)	
					3a	Positive	3 (5.5)	7 (4.0)	0.934
						Negative	52 (94.5)	168 (96.0)	
					3b	Positive	0 (0.0)	0 (0.0)	NA
						Negative	14 (100.0)	20 (100.0)	
					7	Positive	5 (5.7)	7 (3.9)	0.739
						Negative	83 (94.3)	171 (96.1)	
					4sa	Positive	0 (0.0)	0 (0.0)	NA
						Negative	26 (100.0)	42 (100.0)	
					8	Positive	0 (0.0)	0 (0.0)	NA
						Negative	21 (100.0)	79 (100.0)	
					9	Positive	1 (3.8)	1 (2.7)	1.000
						Negative	25 (96.2)	36 (97.3)	
					11	Positive	0 (0.0)	0 (0.0)	NA
						Negative	10 (100.0)	21 (100.0)	
					5	Positive	0 (NA)	0 (0.0)	NA
						Negative	0 (NA)	8 (100.0)	
					6	Positive	0 (0.0)	0 (0.0)	NA
						Negative	2 (100.0)	6 (100.0)	
					Total	Positive	37 (3.0)	39 (1.6)	**0.0069**
						Negative	1,182 (97.0)	2,367 (98.4)	

Pearson’s chi-squared or Fisher’s exact test was used. *p*-Values were two-sided.

AJCC, American Joint Committee on Cancer; JCEC, Japanese Classification of Esophageal Cancer; nCRT, neoadjuvant chemoradiotherapy; nICRT, neoadjuvant immunochemoradiotherapy.

Bold values means there is significant difference between the two groups.

**Figure 2:**
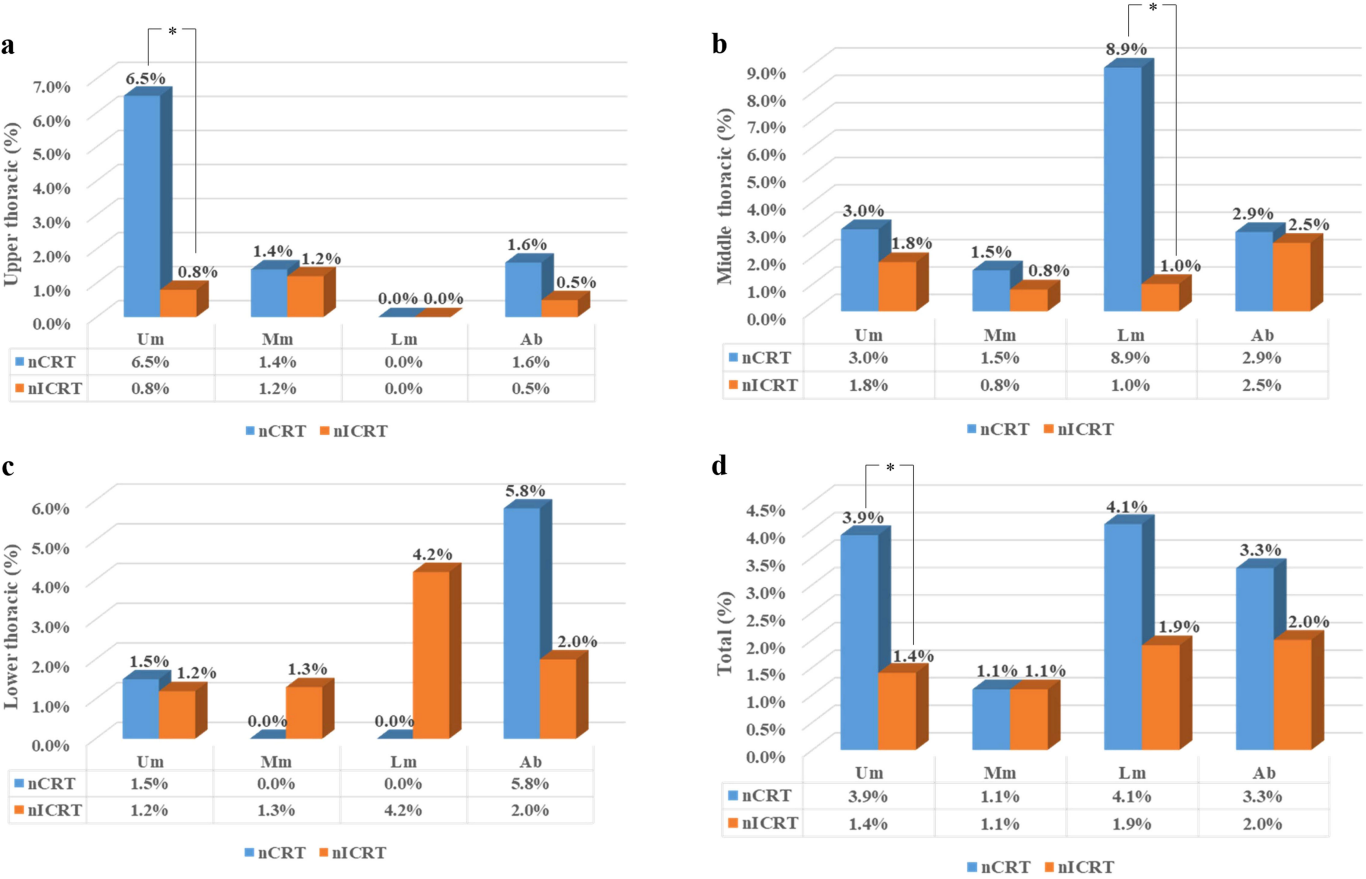
Rates of lymph node metastasis according to the location of the primary tumor. **(a)** Rates of lymph node metastasis in upper thoracic esophageal cancer **(b)** Rates of lymph node metastasis in middle thoracic thoracic esophageal cancer, **(c)** Rates of lymph node metastasis in lower thoracic thoracic esophageal cancer, and **(d)** Rates of lymph node metastasis in all esophageal cancer.nCRT, neoadjuvant chemoradiotherapy; nICRT, neoadjuvant immunochemoradiotherapy; Um, upper mediastinum; Mm, middlemediastinum; Lm, lower mediastinum; Ab, abdomen. *p < 0.05. Pearson’s chi-squared or Fisher’s exact test was used. *p*-Values were two-sided.

**Figure 3:**
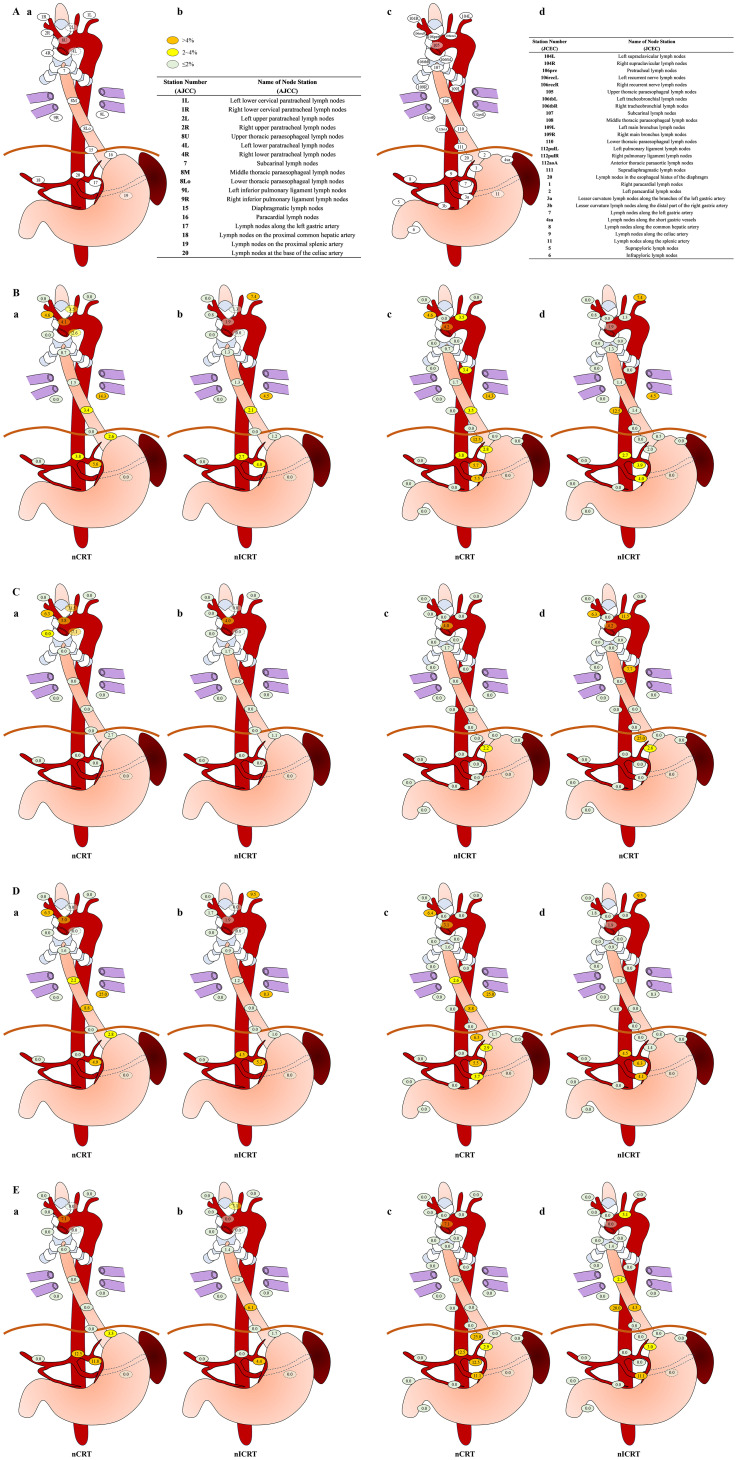
Rates of lymph node metastasis in subgroup analysis. **(A)** Sites and terminology of the regional lymph nodes in esophageal squamous cell carcinoma (ESCC) classified using both eighth edition of American Joint Committee on Cancer (AJCC) esophageal cancer staging system (Ia, Ib) and 11th edition of Japanese Classification of Esophageal Cancer (JCEC) standards (Ic, Id). **(B)** Sites and rates of lymph node metastasis in neoadjuvant chemoradiotherapy (nCRT) (Bb, Bc) and neoadjuvant immunochemoradiotherapy (nICRT) (Bb, Bd) according to both AJCC (Ba, Bb) system and JCEC (Bc, Bd) standards. **(C)** In upper thoracic cases, sites and rates of lymph node metastasis in nCRT (Ca, Cc) and nICRT (Cb, Cd) according to both AJCC (Ca, Cb) system and JCEC (Cc,Cd) standards. **(D)** In middle thoracic cases, sites and rates of lymph node metastasis in nCRT (Da, Dc) and nICRT (Db, Dd) according to both AJCC (Da, Db) system and JCEC (Dc, Dd) standards. **(E)** In lower thoracic cases, sites and rates of lymph node metastasis in nCRT (Ea, Ec) and nICRT (Eb, Ed) according to both AJCC (Ea, Eb) system and JCEC (Ec, Ed) standards. Note. According to the metastasis rate, three different colors are used to label lymph node metastasis, as follows: <2%, light green; 2% to 4%, yellow; >4%, orange. The numbers with decimal points on each labeled lymph node station represent the rates of lymph node metastasis involved in each location. nCRT, neoadjuvant chemoradiotherapy; nICRT, neoadjuvant immunochemoradiotherapy. Pearson’s chi-squared or Fisher’s exact test was used. *p*-Values were two-sided.

Considering that the pattern of lymph node metastasis is related to the location of the tumor, we assessed the stations and frequencies of lymph node metastasis according to tumor location. The rates of lymph node metastasis in the upper mediastinum, middle mediastinum, lower mediastinum, and abdomen in different locations of thoracic ESCC are shown in **Figure 2**. The stations and frequencies of lymph node metastasis in different locations of thoracic ESCC are shown in **Supplementary Tables 1–4** and **Figure 3**. In upper thoracic cases, the rate of upper mediastinal lymph node metastasis in the nICRT group was significantly lower than that in the nCRT group (0.8% vs. 6.5%, *p* = 0.044; **Figure 2**). In middle thoracic cases, the rate of lower mediastinal lymph node metastasis in the nICRT group was significantly lower than that in the nCRT group (1.0% vs. 8.9%, *p* = 0.038; **Figure 2**). The nCRT group had a higher lymph node metastasis rate in station 8Lo of the eighth edition of the AJCC esophageal cancer staging system and station 110 of the 11th JCEC standard compared with the nICRT group (8.8% vs. 0%, *p* = 0.033; **Supplementary Table 2**, **Figure 3**). In lower thoracic cases, the nICRT group had a lower lymph node metastasis rate in station 7 of the 11th JCEC standard compared with the nCRT group (0% vs. 12.5%, *p* = 0.022; **Supplementary Table 3**, **Figure 3**).

## Discussion

Since the publication of two large Phase III trials, the CROSS trial (3) and the NEOCRTEC5010 trial (33), nCRT followed by esophagectomy has become the standardized treatment option for locally advanced ESCC. However, the long-term outcomes of ESCC patients after nCRT and surgery remain poor, with the 5-year cumulative incidence rates of local, distant, and overall recurrence being 15.3%, 24.3%, and 32.2%, respectively (33). Immunotherapy, especially for immune checkpoint inhibitors, has recently emerged as an effective antitumor treatment among various solid tumors, including ESCC. Currently, chemotherapy combined with ICIs has become the standardized treatment for metastatic ESCC patients (34–37). Recently, more and more trials have been performed to investigate the efficacy and toxicity of ICIs in neoadjuvant or definitive radiotherapy settings (38). In our center, we initiated the PALACE-1 trial and demonstrated that the combination of pembrolizumab and nCRT was safe and efficient with a pCR of 55.6% (10/18) (20). We then conducted a multicenter single-arm PALACE-2 trial to validate this new treatment strategy in a large sample size cohort (39). Although neoadjuvant therapy could effectively downstage locally advanced ESCC, standardized lymph node dissection remains important. Previous studies have demonstrated that lymph node status after neoadjuvant treatment and the extent of lymph node dissection were two independent predictors of survival and recurrence in patients with esophageal cancer following neoadjuvant therapy (40). As a result, clarifying the rate of lymph node metastasis at specific stations and distribution patterns after neoadjuvant treatment would provide important information for surgeons to determine the optimal surgical approach and extent of lymph node dissection, which would aid radiation oncologists in designing the dose of neoadjuvant radiotherapy. Prior to the present study, multiple studies had been published to investigate the frequency and distribution pattern of lymph node metastasis after neoadjuvant chemoradiotherapy or chemoimmunotherapy. However, the rate and distribution of lymph node metastasis in ESCC treated with nICRT remain unknown.

In this large prospective cohort, all ESCC patients were treated with standardized nCRT with or without pembrolizumab. The baseline characteristics were comparable between the two groups. Our results showed that the pCR rate of ESCC after nICRT was 44.9%, which was higher than that after nCRT (37%), but lacked statistical significance. The pCR rate was consistent with that of the CROSS trial (49%) and the NEOCRTEC5010 (43.2%) trial (6). As for major pathological response, ESCC treated with nICT (immune checkpoint inhibitors with chemotherapy) varied in pCR, ranging from 16.7% to 50%, and MPR, ranging from 41.7% to 72.2%, in a recent meta-analysis (41), which showed that nICT had no significant advantage according to the pCR and MPR rates when compared to nCRT. In the present study, the nICRT regimen significantly improved MPR rate in comparison to nCRT alone (79.5% vs. 65.4%, *p* = 0.024). Therefore, our results suggested that the addition of ICIs to nCRT improved the tumor regression when compared to nCRT alone. Studies on applying immunotherapy in esophageal cancer have been emerging constantly in recent years. The CheckMate 577 trial has reported the efficacy of adjuvant nivolumab following nCRT in non-pCR patients with esophageal cancer (42). According to the oral report in American Society of Clinical Oncology (ASCO) 2025, among the 532 patients who received nivolumab, the median disease-free survival was 21.8 months [95% confidence interval (CI), 16.6 to 29.7], as compared with 10.8 months (95% CI, 8.3 to 14.3) among the 262 patients who received placebo (hazard ratio for disease recurrence or death, 0.69; 96.4% CI, 0.56 to 0.86; *p* < 0.001). Compared to the clinical significance of postoperative immunotherapy, our regimen, adding pembrolizumab to preoperative CRT, is expected to improve the pCR rates and therefore reduce the tumor burden during surgery, which contributes to a higher R0 resection rate. Furthermore, immunotherapy has a synergistic effect with radiotherapy, and their combined application in nICRT can better improve treatment outcomes.

Among the 208 ESCC patients treated with neoadjuvant therapy followed by surgery, the overall lymph node positivity rate was 20.7%, with 27.2% in the nCRT cohort and 16.5% in the nICRT cohort. The lymph node positivity rate in the present study was significantly lower than that in patients treated with nICT as reported by Zhou H. et al. (42.8%) (43). Sun H. B. et al. (44) reported a lymph node positivity rate of 39.2% among a cohort of 398 ESCC patients treated with neoadjuvant chemoimmunotherapy and surgery. In another trial conducted by Tang H. et al., the lymph node positivity rate was 36.4% (45). There are two possible reasons for this finding: 1) our study was a retrospective cohort study based on a prospective database, and all patients were treated with the same radiotherapy dose and concurrent chemoradiotherapy according to trial protocol; however, these published studies were retrospective research, so selection bias could not be avoided. In addition, the treatment regimen significantly varied, which would be another source of heterogeneity impacting the results; 2) the previous two studies investigated lymph node metastasis in ESCC after neoadjuvant systemic therapy, and they found that the addition of radiotherapy to neoadjuvant systemic therapy could further decrease lymph node metastasis. According to the number of resected lymph nodes, a total of 1,176 and 2,230 lymph nodes had been identified in the present study, with 37 and 39 metastatic lymph nodes in the nCRT and nICRT groups, respectively (3.1% vs. 1.7%, *p* = 0.0069). Based on a prospective database, we found that the overall lymph node positivity rate among ESCC patients treated with nCRT with or without pembrolizumab was relatively low, and the addition of pembrolizumab had a tendency to decrease the risk of lymph node metastasis.

As for the specific station of lymph node metastasis, the common sites of lymph node metastasis after neoadjuvant treatment were station 112pulL (8.3%, 3/36 nodes positive), followed by station 104L (4.9%, 2/41 nodes positive), station 7 (4.5%, 12/266 nodes positive), and station 3a (4.3%, 10/230 nodes positive) according to 11th JCEC standard. Zhou H. et al. (43) found that the common sites of lymph node metastasis included station 107 (12.8%), station 106recR (11.7%), and station 7 (12.5%) after neoadjuvant chemoimmunotherapy. Sun H. B. et al. found that the most common metastatic sites were the right upper paratracheal (16.8%) and left gastric artery (13.1%) stations (44) among ESCC patients after neoadjuvant chemotherapy. Based on our findings, we suggest that different neoadjuvant treatment modalities may result in varying frequencies and distributions of lymph node metastasis. Then, we compared the pattern of lymph node metastasis between nCRT and nICRT and found that the metastasis rate of lymph nodes in station 2R in patients receiving nICRT was significantly lower than that of patients receiving nCRT (0.8% vs. 4.6%, *p* = 0.039). Based on the 11th JCEC standard, the metastasis rates of lymph nodes in station 106recR (0.8% vs. 4.6%, *p* = 0.042) and station 20 (0% vs. 12.5%, *p* = 0.030) had a significant difference between nICRT and nCRT.

The mechanism by which immunotherapy reduces lymph node metastasis in esophageal cancer is a complex, multi-layered, and interconnected process. The core of this approach lies in breaking the immune suppression of tumors and reactivating and guiding the human immune system to recognize, attack, and eliminate primary tumors and metastatic cancer cells in lymph nodes. The immune microenvironment has been shown to play a critical role in the progression of ESCC. Radiotherapy can modulate the immune microenvironment, thereby enhancing the efficacy of immunotherapy. Research has emphasized the importance of tertiary lymphoid structures (TLSs) in the therapeutic efficacy and prognosis of ESCC patients receiving nICRT (46). As for the certain nodal reduction (e.g., 106recR), the recurrent laryngeal nerve lymph node group is the core hub for the upward drainage of lymph from the cervical and upper thoracic esophagus. When tumor cells flow upward with lymph, they will be the first to reach the recurrent laryngeal nerve area (47). This unique anatomical location, extensive lymphatic drainage function, and the lymphatic reflux characteristics determine that patients have a higher risk of metastasis, so they can benefit more from immunotherapy. According to the lymph node metastasis distribution analyzed in our study, patients with preoperative radiologically suspicious lymph node metastasis in the right recurrent nerve area may benefit most from pembrolizumab addition. Patients with tumors located in the upper thoracic segments and radiologically suspicious lymph node metastasis in the upper mediastina, and those with tumors located in the middle thoracic segments and radiologically suspicious lymph node metastasis in the lower mediastina, may benefit from pembrolizumab addition as well. In summary, our study indicated that the common sites of lymph node metastasis significantly varied among ESCC patients after different neoadjuvant treatments, and nICRT could further decrease lymph node metastasis in a specific station in comparison with nCRT.

The strength of the present study was that it was a retrospective study based on a prospective cohort database, and all patients were treated with a standardized nCRT regimen with or without pembrolizumab followed by esophagectomy. All patients were treated with the same radiotherapy dose, concurrent chemotherapy, and pembrolizumab dose. However, our study has several limitations. First, this was a single-center, retrospective study, and the sample size was relatively small; therefore, selection bias could not be avoided. Second, our analysis lacked external validation. Further prospective multicenter clinical trials are recommended to confirm our findings.

In conclusion, in comparison with nCRT, nICRT followed by surgery may lead to a promising pathological response of the primary tumor and decrease the metastasis of lymph nodes. The common sites of lymph node metastasis after neoadjuvant treatment were station 112pulL, followed by station 104L, station 7, and station 3a, according to the 11th JCEC standard. The distribution of metastatic lymph nodes significantly varies according to neoadjuvant treatment strategy and the location of the primary tumor, and nICRT should be considered as a better preoperative treatment option.

## Data Availability

The raw data supporting the conclusions of this article will be made available by the authors, without undue reservation.
