# Supplementary material for: Residual pattern of primary tumor and lymph node in ESCC treated with nCRT with or without pembrolizumab: an analysis from a prospective cohort
Source: Front Immunol. 2025 Oct 22;16:1700400. doi: 10.3389/fimmu.2025.1700400 (PMC12585963; doi:10.3389/fimmu.2025.1700400)
Supplement: Supplementary file 1 [file Table1.docx]

Supplemental Table 1. Lymph node metastasis rates of different stations classified by AJCC and JCEC standard among two groups of patients in upper thoracic cases

| **AJCC** |  |  |  |  |  | **JCEC** |  |  |  |  |
| --- | --- | --- | --- | --- | --- | --- | --- | --- | --- | --- |
| **Station** | **Level** | **nCRT(%)** | **nICRT(%)** | ***P* value** |  | **Station** | **Level** | **nCRT(%)** | **nICRT(%)** | ***P* value** |
| **1L** | Positive | 0(0.0) | 0(0.0) | NA |  | **104L** | Positive | 0(0.0) | 0(0.0) | NA |
|  | Negative | 9(100.0) | 5(100.0) |  |  |  | Negative | 9(100.0) | 5(100.0) |  |
| **1R** | Positive | 0(NA) | 0(0.0) | NA |  | **104R** | Positive | 0(NA) | 0(0.0) | NA |
|  | Negative | 0(NA) | 12(100.0) |  |  |  | Negative | 0(NA) | 12(100.0) |  |
| **2L** | Positive | 3(11.5) | 0(0.0) | 0.376 |  | **106pre** | Positive | 0(NA) | 0(0.0) | NA |
|  | Negative | 23(88.5) | 18(100.0) |  |  |  | Negative | 0(NA) | 3(100.0) |  |
| **2R** | Positive | 3(6.3) | 0(0.0) | 0.151 |  | **106recL** | Positive | 3(11.5) | 0(0.0) | 0.376 |
|  | Negative | 45(93.8) | 64(100.0) |  |  |  | Negative | 23(88.5) | 18(100.0) |  |
| **8U** | Positive | 1(3.8) | 1(4.0) | 0.977 |  | **106recR** | Positive | 3(6.3) | 0(0.0) | 0.164 |
|  | Negative | 25(96.2) | 24(96.0) |  |  |  | Negative | 45(93.8) | 61(100.0) |  |
| **4L** | Positive | 1(7.1) | 0(0.0) | 1.000 |  | **105** | Positive | 1(4.2) | 1(4.0) | 0.976 |
|  | Negative | 13(92.9) | 9(100.0) |  |  |  | Negative | 23(95.8) | 24(96.0) |  |
| **4R** | Positive | 0(0.0) | 0(0.0) | NA |  | **106tbL** | Positive | 0(0.0) | 0(0.0) | NA |
|  | Negative | 6(100.0) | 1(100.0) |  |  |  | Negative | 1(100.0) | 4(100.0) |  |
| **7** | Positive | 0(0.0) | 1(1.7) | 1.000 |  | **106tbR** | Positive | 0(0.0) | 0(NA) | NA |
|  | Negative | 47(100.0) | 57(98.3) |  |  |  | Negative | 3(100.0) | 0(NA) |  |
| **8M** | Positive | 0(0.0) | 0(0.0) | NA |  | **107** | Positive | 0(0.0) | 1(1.7) | 1.000 |
|  | Negative | 7(100.0) | 16(100.0) |  |  |  | Negative | 47(100.0) | 57(98.3) |  |
| **8Lo** | Positive | 0(0.0) | 0(0.0) | NA |  | **108** | Positive | 0(0.0) | 0(0.0) | NA |
|  | Negative | 19(100.0) | 26(100.0) |  |  |  | Negative | 7(100.0) | 16(100.0) |  |
| **9L** | Positive | 0(0.0) | 0(0.0) | NA |  | **109L** | Positive | 1(7.7) | 0(0.0) | 1.000 |
|  | Negative | 3(100.0) | 3(100.0) |  |  |  | Negative | 12(92.3) | 5(100.0) |  |
| **9R** | Positive | 0(NA) | 0(NA) | NA |  | **109R** | Positive | 0(0.0) | 0(0.0) | NA |
|  | Negative | 0(NA) | 0(NA) |  |  |  | Negative | 3(100.0) | 1(100.0) |  |
| **15** | Positive | 0(0.0) | 0(0.0) | NA |  | **110** | Positive | 0(0.0) | 0(0.0) | NA |
|  | Negative | 3(100.0) | 6(100.0) |  |  |  | Negative | 19(100.0) | 26(100.0) |  |
| **16** | Positive | 2(2.7) | 1(1.1) | 0.879 |  | **112pulL** | Positive | 0(0.0) | 0(0.0) | NA |
|  | Negative | 72(97.3) | 87(98.9) |  |  |  | Negative | 3(100.0) | 3(100.0) |  |
| **17** | Positive | 0(0.0) | 0(0.0) | NA |  | **112pulR** | Positive | 0(NA) | 0(NA) | NA |
|  | Negative | 27(100.0) | 76(100.0) |  |  |  | Negative | 0(NA) | 0(NA) |  |
| **18** | Positive | 0(0.0) | 0(0.0) | NA |  | **112aoA** | Positive | 0(0.0) | 0(NA) | NA |
|  | Negative | 5(100.0) | 16(100.0) |  |  |  | Negative | 2(100.0) | 0(NA) |  |
| **19** | Positive | 0(0.0) | 0(0.0) | NA |  | **111** | Positive | 0(0.0) | 0(0.0) | NA |
|  | Negative | 2(100.0) | 1(100.0) |  |  |  | Negative | 3(100.0) | 6(100.0) |  |
| **20** | Positive | 0(0.0) | 0(0.0) | NA |  | **20** | Positive | 1(25.0) | 0(0.0) | 0.286 |
|  | Negative | 9(100.0) | 4(100.0) |  |  |  | Negative | 3(75.0) | 10(100.0) |  |
| **Total** | Positive | 10(3.1) | 3(0.7) | **0.013** |  | **1** | Positive | 1(2.6) | 1(2.2) | 1.000 |
|  | Negative | 315(96.9) | 425(99.3) |  |  |  | Negative | 37(97.4) | 44(97.8) |  |
|  |  |  |  |  |  | **2** | Positive | 0(0.0) | 0(0.0) | NA |
|  |  |  |  |  |  |  | Negative | 32(100.0) | 33(100.0) |  |
|  |  |  |  |  |  | **3a** | Positive | 0(0.0) | 0(0.0) | NA |
|  |  |  |  |  |  |  | Negative | 10(100.0) | 51(100.0) |  |
|  |  |  |  |  |  | **3b** | Positive | 0(0.0) | 0(0.0) | NA |
|  |  |  |  |  |  |  | Negative | 1(100.0) | 6(100.0) |  |
|  |  |  |  |  |  | **7** | Positive | 0(0.0) | 0(0.0) | NA |
|  |  |  |  |  |  |  | Negative | 17(100.0) | 25(100.0) |  |
|  |  |  |  |  |  | **4sa** | Positive | 0(0.0) | 0(0.0) | NA |
|  |  |  |  |  |  |  | Negative | 6(100.0) | 8(100.0) |  |
|  |  |  |  |  |  | **8** | Positive | 0(0.0) | 0(0.0) | NA |
|  |  |  |  |  |  |  | Negative | 5(100.0) | 16(100.0) |  |
|  |  |  |  |  |  | **9** | Positive | 0(0.0) | 0(0.0) | NA |
|  |  |  |  |  |  |  | Negative | 9(100.0) | 4(100.0) |  |
|  |  |  |  |  |  | **11** | Positive | 0(0.0) | 0(0.0) | NA |
|  |  |  |  |  |  |  | Negative | 2(100.0) | 1(100.0) |  |
|  |  |  |  |  |  | **5** | Positive | 0(NA) | 0(NA) | NA |
|  |  |  |  |  |  |  | Negative | 0(NA) | 0(NA) |  |
|  |  |  |  |  |  | **6** | Positive | 0(NA) | 0(NA) | NA |
|  |  |  |  |  |  |  | Negative | 0(NA) | 0(NA) |  |
|  |  |  |  |  |  | **Total** | Positive | 10(3.0) | 3(0.7) | **0.012** |
|  |  |  |  |  |  |  | Negative | 322(97.0) | 439(99.3) |  |
