# Supplementary material for: Residual pattern of primary tumor and lymph node in ESCC treated with nCRT with or without pembrolizumab: an analysis from a prospective cohort
Source: Front Immunol. 2025 Oct 22;16:1700400. doi: 10.3389/fimmu.2025.1700400 (PMC12585963; doi:10.3389/fimmu.2025.1700400)
Supplement: Supplementary file 2 [file Table2.docx]

Supplemental Table 2. Lymph node metastasis rates of different stations classified by AJCC and JCEC standard among two groups of patients in middle thoracic cases

| AJCC |  |  |  |  |  | JCEC |  |  |  |  |
| --- | --- | --- | --- | --- | --- | --- | --- | --- | --- | --- |
| Station | Level | nCRT(%) | nICRT(%) | P value |  | Station | Level | nCRT(%) | nICRT(%) | P value |
| 1L | Positive | 0(0.0) | 2(9.5) | 1.000 |  | 104L | Positive | 0(0.0) | 2(9.5) | 1.000 |
|  | Negative | 5(100.0) | 19(90.5) |  |  |  | Negative | 5(100.0) | 19(90.5) |  |
| 1R | Positive | 0(0.0) | 0(0.0) | NA |  | 104R | Positive | 0(0.0) | 0(0.0) | NA |
|  | Negative | 4(100.0) | 8(100.0) |  |  |  | Negative | 4(100.0) | 8(100.0) |  |
| 2L | Positive | 0(0.0) | 0(0.0) | NA |  | 106pre | Positive | 0(0.0) | 0(0.0) | NA |
|  | Negative | 43(100.0) | 76(100.0) |  |  |  | Negative | 2(100.0) | 7(100.0) |  |
| 2R | Positive | 3(6.3) | 2(1.7) | 0.306 |  | 106recL | Positive | 0(0.0) | 0(0.0) | NA |
|  | Negative | 45(93.8) | 113(98.3) |  |  |  | Negative | 43(100.0) | 76(100.0) |  |
| 8U | Positive | 1(3.0) | 1(1.9) | 1.000 |  | 106recR | Positive | 3(6.4) | 2(1.8) | 0.309 |
|  | Negative | 32(97.0) | 53(98.1) |  |  |  | Negative | 44(93.6) | 110(98.2) |  |
| 4L | Positive | 0(0.0) | 0(0.0) | NA |  | 105 | Positive | 1(3.1) | 1(1.9) | 1.000 |
|  | Negative | 18(100.0) | 42(100.0) |  |  |  | Negative | 31(96.9) | 53(98.1) |  |
| 4R | Positive | 0(0.0) | 0(0.0) | NA |  | 106tbL | Positive | 0(0.0) | 0(0.0) | NA |
|  | Negative | 11(100.0) | 24(100.0) |  |  |  | Negative | 5(100.0) | 5(100.0) |  |
| 7 | Positive | 1(1.6) | 1(0.9) | 1.000 |  | 106tbR | Positive | 0(0.0) | 0(NA) | NA |
|  | Negative | 63(98.4) | 109(99.1) |  |  |  | Negative | 2(100.0) | 0(NA) |  |
| 8M | Positive | 1(2.2) | 1(1.2) | 1.000 |  | 107 | Positive | 1(1.6) | 1(0.9) | 1.000 |
|  | Negative | 44(97.8) | 84(98.8) |  |  |  | Negative | 63(98.4) | 109(99.1) |  |
| 8Lo | Positive | 3(8.8) | 0(0.0) | 0.033 |  | 108 | Positive | 1(2.6) | 1(1.2) | 0.543 |
|  | Negative | 31(91.2) | 70(100.0) |  |  |  | Negative | 38(97.4) | 81(98.8) |  |
| 9L | Positive | 2(25.0) | 1(8.3) | 0.701 |  | 109L | Positive | 0(0.0) | 0(0.0) | NA |
|  | Negative | 6(75.0) | 11(91.7) |  |  |  | Negative | 13(100.0) | 37(100.0) |  |
| 9R | Positive | 0(0.0) | 0(0.0) | NA |  | 109R | Positive | 0(0.0) | 0(0.0) | NA |
|  | Negative | 5(100.0) | 9(100.0) |  |  |  | Negative | 9(100.0) | 20(100.0) |  |
| 15 | Positive | 0(0.0) | 0(0.0) | NA |  | 110 | Positive | 3(8.8) | 0(0.0) | 0.033 |
|  | Negative | 3(100.0) | 9(100.0) |  |  |  | Negative | 31(91.2) | 70(100.0) |  |
| 16 | Positive | 4(2.8) | 3(1.0) | 0.341 |  | 112pulL | Positive | 2(25.0) | 1(8.3) | 0.701 |
|  | Negative | 141(97.2) | 289(99.0) |  |  |  | Negative | 6(75.0) | 11(91.7) |  |
| 17 | Positive | 4(4.9) | 11(5.3) | 1.000 |  | 112pulR | Positive | 0(0.0) | 0(0.0) | NA |
|  | Negative | 78(95.1) | 198(94.7) |  |  |  | Negative | 5(100.0) | 9(100.0) |  |
| 18 | Positive | 0(0.0) | 0(0.0) | NA |  | 112aoA | Positive | 0(0.0) | 0(0.0) | NA |
|  | Negative | 10(100.0) | 38(100.0) |  |  |  | Negative | 6(100.0) | 3(100.0) |  |
| 19 | Positive | 0(0.0) | 0(0.0) | NA |  | 111 | Positive | 0(0.0) | 0(0.0) | NA |
|  | Negative | 7(100.0) | 16(100.0) |  |  |  | Negative | 3(100.0) | 9(100.0) |  |
| 20 | Positive | 0(0.0) | 1(4.5) | 1.000 |  | 20 | Positive | 1(6.3) | 0(0.0) | 0.364 |
|  | Negative | 9(100.0) | 21(95.5) |  |  |  | Negative | 15(93.8) | 28(100.0) |  |
| Total | Positive | 19(3.3) | 23(1.9) | 0.066 |  | 1 | Positive | 2(2.9) | 2(1.4) | 0.869 |
|  | Negative | 555(96.7) | 1189(98.1) |  |  |  | Negative | 68(97.1) | 136(98.6) |  |
|  |  |  |  |  |  | 2 | Positive | 1(1.7) | 1(0.8) | 0.537 |
|  |  |  |  |  |  |  | Negative | 58(98.3) | 125(99.2) |  |
|  |  |  |  |  |  | 3a | Positive | 1(3.7) | 4(4.1) | 1.000 |
|  |  |  |  |  |  |  | Negative | 26(96.3) | 93(95.9) |  |
|  |  |  |  |  |  | 3b | Positive | 0(0.0) | 0(0.0) | NA |
|  |  |  |  |  |  |  | Negative | 8(100.0) | 14(100.0) |  |
|  |  |  |  |  |  | 7 | Positive | 3(5.5) | 7(6.3) | 1.000 |
|  |  |  |  |  |  |  | Negative | 52(94.5) | 105(93.8) |  |
|  |  |  |  |  |  | 4sa | Positive | 0(0.0) | 0(0.0) | NA |
|  |  |  |  |  |  |  | Negative | 15(100.0) | 19(100.0) |  |
|  |  |  |  |  |  | 8 | Positive | 0(0.0) | 0(0.0) | NA |
|  |  |  |  |  |  |  | Negative | 10(100.0) | 38(100.0) |  |
|  |  |  |  |  |  | 9 | Positive | 0(0.0) | 1(4.5) | 1.000 |
|  |  |  |  |  |  |  | Negative | 9(100.0) | 21(95.5) |  |
|  |  |  |  |  |  | 11 | Positive | 0(0.0) | 0(0.0) | NA |
|  |  |  |  |  |  |  | Negative | 7(100.0) | 16(100.0) |  |
|  |  |  |  |  |  | 5 | Positive | 0(NA) | 0(0.0) | NA |
|  |  |  |  |  |  |  | Negative | 0(NA) | 2(100.0) |  |
|  |  |  |  |  |  | 6 | Positive | 0(NA) | 0(NA) | NA |
|  |  |  |  |  |  |  | Negative | 0(NA) | 0(NA) |  |
|  |  |  |  |  |  | Total | Positive | 19(3.2) | 23(1.8) | 0.072 |
|  |  |  |  |  |  |  | Negative | 578(96.8) | 1224(98.2) |  |
