# Supplementary material for: Residual pattern of primary tumor and lymph node in ESCC treated with nCRT with or without pembrolizumab: an analysis from a prospective cohort
Source: Front Immunol. 2025 Oct 22;16:1700400. doi: 10.3389/fimmu.2025.1700400 (PMC12585963; doi:10.3389/fimmu.2025.1700400)
Supplement: Supplementary file 3 [file Table3.docx]

Supplemental Table 3. Lymph node metastasis rates of different stations classified by AJCC and JCEC standard among two groups of patients in lower thoracic cases

| **AJCC** |  |  |  |  |  | **JCEC** |  |  |  |  |
| --- | --- | --- | --- | --- | --- | --- | --- | --- | --- | --- |
| **Station** | **Level** | **nCRT(%)** | **nICRT(%)** | ***P* value** |  | **Station** | **Level** | **nCRT(%)** | **nICRT(%)** | ***P* value** |
| **1L** | Positive | 0(NA) | 0(0.0) | NA |  | **104L** | Positive | 0(NA) | 0(0.0) | NA |
|  | Negative | 0(NA) | 1(100.0) |  |  |  | Negative | 0(NA) | 1(100.0) |  |
| **1R** | Positive | 0(NA) | 0(NA) | NA |  | **104R** | Positive | 0(NA) | 0(NA) | NA |
|  | Negative | 0(NA) | 0(NA) |  |  |  | Negative | 0(NA) | 0(NA) |  |
| **2L** | Positive | 0(0.0) | 2(3.1) | 0.345 |  | **106pre** | Positive | 0(0.0) | 0(0.0) | NA |
|  | Negative | 16(100.0) | 63(96.9) |  |  |  | Negative | 1(100.0) | 5(100.0) |  |
| **2R** | Positive | 0(0.0) | 0(0.0) | NA |  | **106recL** | Positive | 0(0.0) | 2(3.1) | 0.345 |
|  | Negative | 35(100.0) | 70(100.0) |  |  |  | Negative | 16(100.0) | 63(96.9) |  |
| **8U** | Positive | 1(7.1) | 0(0.0) | 0.341 |  | **106recR** | Positive | 0(0.0) | 0(0.0) | NA |
|  | Negative | 13(92.9) | 27(100.0) |  |  |  | Negative | 35(100.0) | 70(100.0) |  |
| **4L** | Positive | 0(0.0) | 0(0.0) | NA |  | **105** | Positive | 1(7.1) | 0(0.0) | 0.341 |
|  | Negative | 7(100.0) | 24(100.0) |  |  |  | Negative | 13(92.9) | 27(100.0) |  |
| **4R** | Positive | 0(0.0) | 0(0.0) | NA |  | **106tbL** | Positive | 0(0.0) | 0(0.0) | NA |
|  | Negative | 2(100.0) | 14(100.0) |  |  |  | Negative | 4(100.0) | 10(100.0) |  |
| **7** | Positive | 0(0.0) | 2(1.4) | 0.322 |  | **106tbR** | Positive | 0(NA) | 0(0.0) | NA |
|  | Negative | 40(100.0) | 143(98.6) |  |  |  | Negative | 0(NA) | 4(100.0) |  |
| **8M** | Positive | 0(0.0) | 1(2.0) | 1.000 |  | **107** | Positive | 0(0.0) | 2(1.4) | 0.322 |
|  | Negative | 14(100.0) | 50(98.0) |  |  |  | Negative | 40(100.0) | 143(98.6) |  |
| **8Lo** | Positive | 0(0.0) | 3(6.1) | 0.371 |  | **108** | Positive | 0(0.0) | 1(2.1) | 1.000 |
|  | Negative | 35(100.0) | 46(93.9) |  |  |  | Negative | 14(100.0) | 47(97.9) |  |
| **9L** | Positive | 0(0.0) | 0(0.0) | NA |  | **109L** | Positive | 0(0.0) | 0(0.0) | NA |
|  | Negative | 3(100.0) | 7(100.0) |  |  |  | Negative | 3(100.0) | 14(100.0) |  |
| **9R** | Positive | 0(NA) | 0(0.0) | NA |  | **109R** | Positive | 0(0.0) | 0(0.0) | NA |
|  | Negative | 0(NA) | 4(100.0) |  |  |  | Negative | 1(100.0) | 5(100.0) |  |
| **15** | Positive | 0(0.0) | 0(0.0) | NA |  | **110** | Positive | 0(0.0) | 2(4.3) | 0.141 |
|  | Negative | 2(100.0) | 8(100.0) |  |  |  | Negative | 33(100.0) | 45(95.7) |  |
| **16** | Positive | 2(3.3) | 2(1.7) | 0.878 |  | **112pulL** | Positive | 0(0.0) | 0(0.0) | NA |
|  | Negative | 58(96.7) | 115(98.3) |  |  |  | Negative | 3(100.0) | 7(100.0) |  |
| **17** | Positive | 4(11.8) | 3(4.4) | 0.332 |  | **112pulR** | Positive | 0(NA) | 0(0.0) | NA |
|  | Negative | 30(88.2) | 65(95.6) |  |  |  | Negative | 0(NA) | 4(100.0) |  |
| **18** | Positive | 0(0.0) | 0(0.0) | NA |  | **112aoA** | Positive | 0(0.0) | 1(20.0) | 1.000 |
|  | Negative | 6(100.0) | 25(100.0) |  |  |  | Negative | 2(100.0) | 4(80.0) |  |
| **19** | Positive | 0(0.0) | 0(0.0) | NA |  | **111** | Positive | 0(0.0) | 0(0.0) | NA |
|  | Negative | 1(100.0) | 4(100.0) |  |  |  | Negative | 2(100.0) | 8(100.0) |  |
| **20** | Positive | 1(12.5) | 0(0.0) | 0.421 |  | **20** | Positive | 1(25.0) | 0(0.0) | 0.235 |
|  | Negative | 7(87.5) | 11(100.0) |  |  |  | Negative | 3(75.0) | 13(100.0) |  |
| **Total** | Positive | 8(2.9) | 13(1.9) | 0.333 |  | **1** | Positive | 1(2.9) | 2(3.0) | 1.000 |
|  | Negative | 269(97.1) | 677(98.1) |  |  |  | Negative | 34(97.1) | 65(97.0) |  |
|  |  |  |  |  |  | **2** | Positive | 0(0.0) | 0(0.0) | NA |
|  |  |  |  |  |  |  | Negative | 21(100.0) | 37(100.0) |  |
|  |  |  |  |  |  | **3a** | Positive | 2(11.1) | 3(11.1) | 1.000 |
|  |  |  |  |  |  |  | Negative | 16(88.9) | 24(88.9) |  |
|  |  |  |  |  |  | **3b** | Positive | 0(0.0) | 0(NA) | NA |
|  |  |  |  |  |  |  | Negative | 5(100.0) | 0(NA) |  |
|  |  |  |  |  |  | **7** | Positive | 2(12.5) | 0(0.0) | **0.022** |
|  |  |  |  |  |  |  | Negative | 14(87.5) | 41(100.0) |  |
|  |  |  |  |  |  | **4sa** | Positive | 0(0.0) | 0(0.0) | NA |
|  |  |  |  |  |  |  | Negative | 5(100.0) | 15(100.0) |  |
|  |  |  |  |  |  | **8** | Positive | 0(0.0) | 0(0.0) | NA |
|  |  |  |  |  |  |  | Negative | 6(100.0) | 25(100.0) |  |
|  |  |  |  |  |  | **9** | Positive | 1(12.5) | 0(0.0) | 0.421 |
|  |  |  |  |  |  |  | Negative | 7(87.5) | 11(100.0) |  |
|  |  |  |  |  |  | **11** | Positive | 0(0.0) | 0(0.0) | NA |
|  |  |  |  |  |  |  | Negative | 1(100.0) | 4(100.0) |  |
|  |  |  |  |  |  | **5** | Positive | 0(NA) | 0(0.0) | NA |
|  |  |  |  |  |  |  | Negative | 0(NA) | 6(100.0) |  |
|  |  |  |  |  |  | **6** | Positive | 0(0.0) | 0(0.0) | NA |
|  |  |  |  |  |  |  | Negative | 2(100.0) | 6(100.0) |  |
|  |  |  |  |  |  | **Total** | Positive | 8(2.8) | 13(1.8) | 0.338 |
|  |  |  |  |  |  |  | Negative | 281(97.2) | 704(98.2) |  |
